# Targeting Treatment Resistance in Head and Neck Squamous Cell Carcinoma – Proof of Concept for CT Radiomics-Based Identification of Resistant Sub-Volumes

**DOI:** 10.3389/fonc.2021.664304

**Published:** 2021-05-27

**Authors:** Marta Bogowicz, Matea Pavic, Oliver Riesterer, Tobias Finazzi, Helena Garcia Schüler, Edna Holz-Sapra, Leonie Rudofsky, Lucas Basler, Manon Spaniol, Andreas Ambrusch, Martin Hüllner, Matthias Guckenberger, Stephanie Tanadini-Lang

**Affiliations:** ^1^ Department of Radiation Oncology, University Hospital Zurich, University of Zurich, Zurich, Switzerland; ^2^ Centre for Radiation Oncology KSA-KSB, Cantonal Hospital Aarau, Aarau, Switzerland; ^3^ Department of Nuclear Medicine, University Hospital Zurich, University of Zurich, Zurich, Switzerland

**Keywords:** local radiomics, radioresistance, head and neck cancer, predictive biomarker, contrast-enhanced CT, tumor recurrence

## Abstract

**Purpose:**

Radiomics has already been proposed as a prognostic biomarker in head and neck cancer (HNSCC). However, its predictive power in radiotherapy has not yet been studied. Here, we investigated a local radiomics approach to distinguish between tumor sub-volumes with different levels of radiosensitivity as a possible target for radiation dose intensification.

**Materials and Methods:**

Of 40 patients (n=28 training and n=12 validation) with biopsy confirmed locally recurrent HNSCC, pretreatment contrast-enhanced CT images were registered with follow-up PET/CT imaging allowing identification of controlled (GTVcontrol) vs non-controlled (GTVrec) tumor sub-volumes on pretreatment imaging. A bi-regional model was built using radiomic features extracted from pretreatment CT in the GTVrec and GTVcontrol to differentiate between those regions. Additionally, concept of local radiomics was implemented to perform detection task. The original tumor volume was divided into sub-volumes with no prior information on the location of recurrence. Radiomic features from those sub-volumes were then used to detect recurrent sub-volumes using multivariable logistic regression.

**Results:**

Radiomic features extracted from non-controlled regions differed significantly from those in controlled regions (training AUC = 0.79 CI 95% 0.66 - 0.91 and validation AUC = 0.88 CI 95% 0.72 – 1.00). Local radiomics analysis allowed efficient detection of non-controlled sub-volumes both in the training AUC = 0.66 (CI 95% 0.56 – 0.75) and validation cohort 0.70 (CI 95% 0.53 – 0.86), however performance of this model was inferior to bi-regional model. Both models indicated that sub-volumes characterized by higher heterogeneity were linked to tumor recurrence.

**Conclusion:**

Local radiomics is able to detect sub-volumes with decreased radiosensitivity, associated with location of tumor recurrence in HNSCC in the pre-treatment CT imaging. This proof of concept study, indicates that local CT radiomics can be used as predictive biomarker in radiotherapy and potential target for dose intensification.

## Introduction

Head and neck squamous cell carcinoma (HNSCC) accounts for approximately 4% of all malignancies across Europe and the USA (1, 2). For locally advanced HNSCC, the standard of care is definitive radiotherapy, whenever possible combined with concurrent chemotherapy. Despite advances in treatment using modern radiotherapy delivery techniques, local recurrences still occur in up to 50% of patients and pose the predominant pattern of failure in HNSCC (3). Therapeutic options for recurrent HNSCC are mainly palliative and comprise salvage surgery, re-irradiation and systemic therapy – however, outcome is poor, and treatment often is associated with significant morbidity (4). Therefore, improvement of primary radiochemotherapy is essential.

Several early clinical trials have studied the role of radiation dose intensification to the primary tumor and metastatic lymph nodes in order to improve local control for HNSCC (5–7). However, concern still exists about excessive acute and late toxicities of this approach and therefore no large randomized trial has been conducted so far. In addition, intensification of radiotherapy by using altered fractionation schemes only lead to very modest improvement of outcome (8). Thus, a dose of 70 Gy delivered over 7 weeks to the entire tumor is widely considered a standard in patients undergoing chemoradation for HNSCC (9). Heterogeneity within a tumor has been recognized as a characteristic of head and neck cancer (10), with some sub-regions being more resistant to radiochemotherapy. Consequently, the strategy of treatment intensification to these sub-volumes could lead to better outcomes in terms of local control and subsequently overall survival, without a significant increase in treatment-related toxicities. Recent advancement in radiation technology in principle allows such an escalation of radiation dose to tumor sub-volumes. However, identification of these sub-volumes is a crucial step within this therapeutic concept.

In contrast to selective biopsy specimens obtained from a small area of the tumor, the use of imaging as biomarker has the advantage to analyze the entire three dimensional tumor volume. The feasibility of delivering a dose boost, so-called dose painting, to tumor sub-volumes has been previously demonstrated, mostly based on functional, metabolism-related (fluorodeoxyglucose) or hypoxia-related (fluoromisonidazole) positron emission tomography (PET) imaging (11–13). In recent years, high-throughput, multidimensional and quantitative images analysis (radiomics) revealed that relevant biological information can be extracted also from routinely acquired, easy to perform morphological imaging, such as regular computed tomography (CT) (14–16). Many investigations have studied the potential of radiomics to predict the risk for metastatic spread, progression-free survival, overall survival or biological phenotypes (17–19) showing encouraging results. In the context of radiotherapy, several studies indicated an association between CT heterogeneity and local tumor control (20–24). However, there is so far scarce data on radiomics as a method to individually tailor dose distribution to specific sub-volumes within a tumor (25–27).

Here, we investigated CT radiomics for identification of radioresistant sub-volumes of the primary tumor leading to persistence or recurrence after curative radiochemotherapy. Reliable detection of these resistant sub-volumes may allow for a tailored treatment by increasing radiation dose to these parts.

## Methods

### Study Population

We retrospectively analyzed patients with primary locally advanced HNSCC (cT3/4 or cN+) treated at our institution between June 2004 and October 2015, who experienced a local in-field tumor recurrence. Local in-field tumor recurrence was defined as a recurrence occurring within the high-dose target volume (excluding lymph nodes), and had to be confirmed by biopsy. Only patients that received a definitive high dose radiation treatment with an equivalent total dose of 68 - 70 Gy in 2-2.11 Gy fractions and a concomitant systemic therapy with either cisplatin and/or cetuximab during the course of radiotherapy were included. A further requirement was a contrast-enhanced CT (CE-CT) imaging before treatment and a FDG-PET/CT at the time of local recurrence. Contrast protocol was not standardized, however patients with contrast visible only in thyroid were not included. Follow up of patients was done according to the institutional routine. The first FDG-PET/CT for treatment response was usually done three months after completion of treatment. A subset of patients included in this study and now reviewed retrospectively has been treated within a prospective trial back then (28).

Data analysis was approved by the Swissethics and was carried out in accordance with Swissethics guidelines and regulations. Patients gave informed general consent.

We identified in total 66 patients fulfilling the above mentioned inclusion criteria. However, twenty-one cases had to be excluded, as a reliable image registration between follow-up ^18^F-fluorodeoxyglucose (^18^F-FDG) PET and CE pretreatment planning CT was not possible. Five additional patients were excluded due to the small size of recurrence (volume < 27 voxels), which precludes a reasonable radiomics analysis. Thus, in total 40 patients were available and were split into a training cohort, consisting of retrospectively collected data (n = 28), and a validation cohort, comprising patients from a prospective phase II clinical study (n = 12) (28). In the majority of cases, the primary tumor was located in the oropharynx, for further details see **Table 1**.

**Table 1:** Patient characteristics of all included patients.

	Training Cohort (n=28)	Validation Cohort (n=12)
**Tumor location**		
Oropharynx	19 (68%)	6 (50%)
Hypopharynx	5 (18%)	4 (33%)
Larynx	3 (11%)	0 (0%)
Oral Cavity	1 (3%)	2 (17%)
**Time to recurrence** (median [range] months)	7 [2 - 59]	8 [4 -24]
**T stage**		
1	1 (3%)	0 (0%)
2	5 (18%)	0 (0%)
3	7 (25%)	3 (25%)
4	15 (54%)	9 (75%)
**N stage**		
0	5 (18%)	2 (17%)
1	4 (14%)	1 (8%)
2a	0 (0%)	1 (8%)
2b	7 (25%)	2 (17%)
2c	12 (43%)	6 (50%)
3	0 (0%)	0 (0%)
**HPV status**		
Positive	3 (11%)	3 (25%)
Negative	12 (43%)	8 (67%)
Unknown	13 (46%)	1 (8%)

### Contrast-Enhanced Planning CT Image Acquisition

Iodine contrast was injected intravenously prior to CT imaging. The contrast protocol varied over the years of data acquisition. Images were acquired on five different scanners (Siemens Somatom Definition AS n = 20, Siemens Volume Zoom n = 13, Siemens Somatom Plus n = 5, GE Healthcare, Discovery STE n = 1 and GE Healthcare, Discovery LS n = 1) with 120 or 140 kV tube voltage, 2 – 3.27mm slice thickness and <1mm in-plane voxel size. The tube current varied between 120-450 mAs, and angular exposure adaption was applied in 7 scans (17%). Images were reconstructed using filtered back projection and soft kernel (B30).

### Definition of Primary Tumor and Recurrence Volume

The primary tumor was contoured based on the CE planning CT assisted by the co-registered pretreatment diagnostic FDG-PET/CT and MRI scans if available. However, in contrary to the FDG-PET, which was available in all the patients, a pretreatment MRI was only present in 5 cases. The recurrence region was contoured as high FDG uptake in the follow-up FDG-PET/CT at the time of the detection of the recurrence (**Supplementary Figure S1**). High FDG uptake region was considered as 40% SUVmax threshold-based sub-volume in the recurrence PET-CT. Details of the PET scanning protocol can be found in the supplement (**Supplementary Table S1**). The contours were then transferred to the initial planning CT by rigid registration with focus on the tumor region (Eclipse v.15, Varian Medical Systems, USA). Two structures were created for further analysis: the overlap of tumor recurrence and primary tumor (GTV_rec_) and the primary tumor contour minus the recurrence (GTV_control_). Prior to radiomics analysis both GTV_rec_ and GTV_control_ were postprocessed, by removal of contours on slices affected by metal artifacts.

### Radiomics Analysis for Differentiation of Controlled to Non-Controlled Tumor Sub-Volumes and Detection of Radioresistant Volumes

Radiomics analysis was performed using Z-Rad software implementation (Python v 2.7). This implementation was benchmarked in the Image Biomarker Standardization Initiative (29, 30). Images were resampled to cubic voxels (3.3 mm, largest voxel dimension in the dataset) using trilinear interpolation. Hounsfield unit range of -20 to 180 HU was set to limit the analysis to soft tissue. Due to the small size of analyzed volumes, the feature set was limited to intensity (n = 25) and texture features (n = 136). The full list of analyzed features is presented in the **Supplementary Table S2**. Volumes with less than 27 voxels were excluded from analysis.

**Figure 1:**
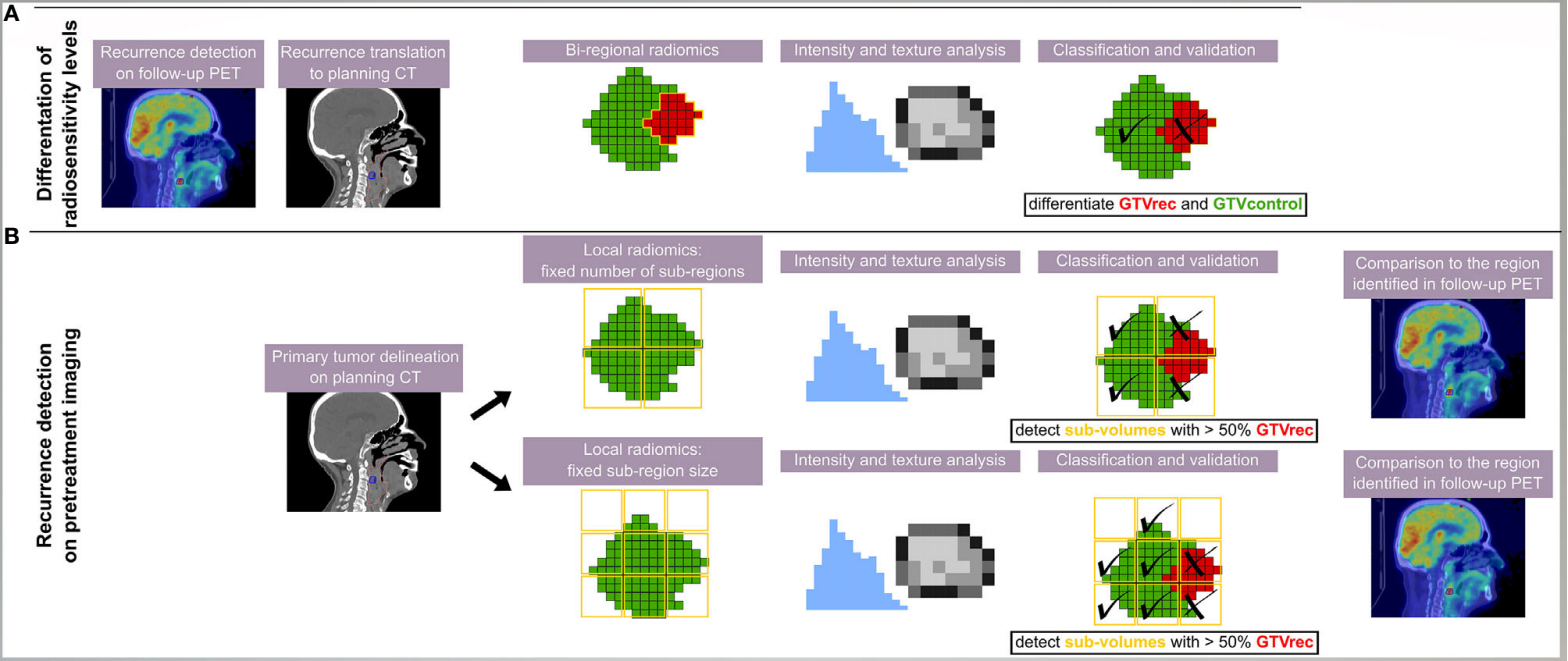
Scheme of the analysis giving an overview on all three radiomics models. The recurrence region was identified on PET/CT imaging and rigidly transferred to the contrast-enhanced planning CT. Different models were trained using different methods and aiming at different purposes. **(A)** In the bi-regional radiomics, features were extracted from GTV_rec_ and GTV_control_ and only a differentiation between recurrent and controlled sub-volumes was performed; **(B)** in two local radiomics models, a detection task was performed and thus sub-volumes were defined without any prior information on the location of recurrence. In the classification task, a sub-volume was classified as recurrence (X) if more than 50% of the voxels belonged to the original contour of the recurrence (red).

In total, three different radiomics models were built (**Figure 1**): The “bi-regional radiomics” analysis aims to differentiate tumor regions, which stay controlled after treatment (GTV_control_) and the non-controlled region, which resulted in a recurrence detected on the FDG-PET CT (GTV_rec_). For detection of radioresistant volumes within the primary tumor without prior knowledge on their location and prior to any treatment two “local radiomics models” were built. Here, the predefined region of interest (ROI), corresponding to the primary GTV and from now on named GTV, is divided into smaller sub-regions, which are later used as a mask for feature extraction. As a consequence, instead of one vector of features describing the GTV, local radiomics returns a set of radiomics-based parametric maps. These maps visualize for example changes in heterogeneity or contrast across the GTV. The local features are calculated based only on the voxels within the GTV, whereas the neighboring voxels (e.g. healthy tissue, manually excluded artifacts) are set to ‘not a number’ (discarded). We have implemented two approaches for the definition of sub-regions (**Figure 1**):

- Division of the GTV into a fixed number of sub-regions with the center of the grid attached to the center of the GTV (in this study 8 sub-regions, the number was chosen to provide sufficient information in 3D and to ensure large enough neighborhood to define texture). The same number of sub-regions was analyzed per patient and their volume depended on the GTV size.
- Division of the GTV into sub-regions with fixed size. In the second approach, the placement of the grid is optimized to cover a GTV volume as large as possible. The full coverage is rarely feasible, because in order to avoid meaningless features calculated only on a few voxels, we have set a threshold T = 25 of minimum number of ROI voxels (voxels with value different than ‘not a number’) in the sub-region. In this study, we decided for 5x5x5 voxels grid with 5 voxel shift. There was no overlap between sub-regions to ensure independent description of the sub-regions. The 5 voxels grid size was a trade-off between ensuring a large enough neighborhood to define texture and a high spatial resolution of the model. Other sizes of the grid were not tested. In contrary to the first approach different numbers of sub-regions were analyzed per patient but their size was constant.

### Statistical Analysis

The classification and detection tasks were performed using logistic regression. In the bi-regional model, the two labels were assigned to GTV_control_ and GTV_rec_ volumes. For local radiomics models, the recurrent label was assigned to regions with more than 50% of voxels overlapping with GTV_rec_. The 50% threshold was chosen arbitrarily, other thresholds were not tested. The remaining sub-regions were labeled as control.

The following feature reduction and binary classification procedure was used. Features with high and moderate correlation to number of voxels in the local region were excluded (r > 0.5). Principal component analysis combined with univariate logistic regression was used for dimensionality reduction (20). The number of retained principal components was computed using the Horn method. Radiomic features were grouped based on their Spearman correlation to principal components. Per group, only the feature with the largest area under operator receiver characteristic curve (AUC) was chosen, given that p-value < 0.05. The final model was built in the multivariable logistic regression with backward selection of variables based on the Akaike Information Criterion. The final model was tested in the separate set as described in the sections below. The confidence intervals were computed with 2000 stratified bootstrap replicates.

For the bi-regional radiomics model and the first local radiomics model based on 8 sub-volumes, training was performed on the training cohort and the results validated in the validation cohort as previously specified in the `study population` section. The 75th percentile threshold of predictions in the training cohort was set as classification threshold to optimize the sensitivity and specificity of the model.

The second local radiomics model with a fixed 5x5x5 grid size, was trained and tested in leave-one-out cross-validation (LOOCV) on the patient level. The full split into training and validation cohort was not feasible due to the presence of metal artifacts in some images. These artifacts, especially when present in the middle of the GTV, strongly influence optimal placement of the grid and thus for this experiment only patients with no visible artifacts in the GTV were selected. Data from 23 patients with no visible CT artifacts were analyzed. The final model was trained on 12 patients (= 48%), who had both classes of sub-volumes (at least one sub-region with 50% contribution of recurrent voxels), which was a requirement of LOOCV.

## Results

### Differentiation of Controlled to Non-Controlled Tumor Regions

**Figure 2:**
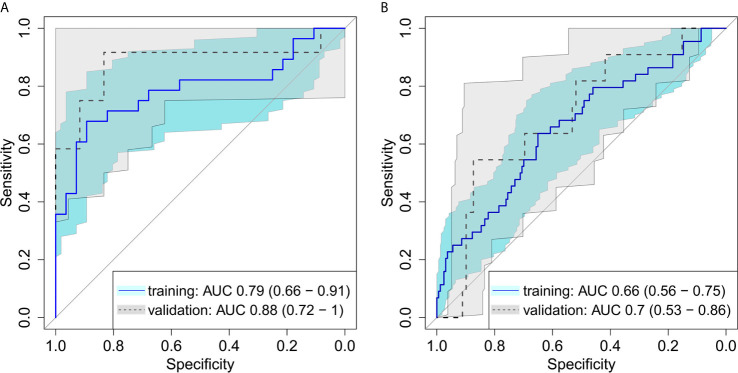
Receiver operating characteristic for **(A)** differentiation between recurrent and controlled sub-volumes in bi-regional radiomics model showing a good discrimination between the radioresistance levels, in both the training and the validation cohort **(B)** detection of recurrent sub-volumes with local radiomics showing a good performance of the model with ability to detect radioresistant sub-volumes of the tumor on pretreatment CT images.

On average, the volume of GTV_rec_ was 20% (range: 2%-71%) of the initial primary tumor volume. A logistic regression model with backward selection of variables showed a good discrimination between GTV_rec_ and GTV_control_, in both the training and the validation cohort, see **Figure 2A** (training AUC = 0.79 (95%CI: 0.66 – 0.91); validation AUC = 0.88 (95%CI: 0.72 – 1.00). This bi-regional radiomics model consisted of two features: “GLRLM gray level non-uniformity” and “merged GLCM sum entropy” - indicating that sub-volumes showing higher density heterogeneity have a higher propensity of recurrence. In the validation cohort, the model achieved high sensitivity (= 0.75) and specificity (= 0.83).

### Detection of Radioresistant Sub-Volumes – Local Radiomics Model

The first model with division of the GTV into 8 equi-volume regions comprised three features: “GLCM cluster shade”, “GLDZM gray level variance” and “histogram median”. This local radiomics model showed slightly inferior performance (AUC = 0.70; 95%CI: 0.53–0.86) in the validation cohort compared to the bi-regional radiomics model (**Figure 2B**). In the validation cohort, in all patients in whom recurrence sub-volumes were detected, at least one of the sub-volumes was correctly identified. The median size of detected recurrence was 43% of the entire recurrence volume. This result is linked to the threshold for recurrence sub-volume definition (50%) and thus can be further improved in the future studies.

For the second definition with division of the GTV into sub-volumes of an equal size (5x5x5 voxels) the rigid grid placement was chosen on an individual patient basis in order to maximize the coverage of studied tumor. 12 patients (= 48%) had both classes of sub-volumes (recurrence and tumor control, based on a 50% criterion). The average AUC in leave-one-out cross-validation was 0.68. In three cases the AUC was below 0.5, indicating worse than random prediction. This was tracked back to a small number of analyzed sub-volumes (e.g. only two sub-volumes) or a high recurrence involvement (more than 25%) in most of the sub-volumes, meaning that in this tumor no true controls with no contribution of radioresistant clones were present. Similar features were selected in different cross validation folds: GLSZM zone size entropy (n=12), GLCM joint maximum (n=9) and histogram range (n=8).

Detailed summary of the all models together with numbers of sub-volumes used for model training and validation is presented in **Table 2**. Number of sub-volumes in the local radiomics analysis per patient is summarized in the **Supplementary Figures S2** and **S3**.

**Table 2:** Details of the final radiomics models.

Model	Model endpoint	AUC training	AUC validation	Selected features	Model coefficients	No of analyzed sub-volumes/No of recurrent sub-volumes	
						training	validation
**Bi-regional radiomics**	Sub-volumes differentiation	0.79 (0.66-0.91)	0.88 (0.72 – 1.00)	GLRLM gray level non-uniformity merged GLCM sum entropy intercept	141.43 4.56 -32.64	56/28	24/12
**Local radiomics:** **fixed number of sub-volumes**	Recurrence detection	0.66 (0.56 – 0.75)	0.70 (0.53 – 0.86)	GLCM cluster shade GLDZM gray level variance histogram median intercept	0.0015 0.019 -0.11 -0.24	222/48	91/11
**Local radiomics:** **fixed size of sub-volumes**	Recurrence detection	—	0.68 (AUC < 0.5 in n=3 cases)	GLSZM zone size entropy*, GLCM joint maximum* histogram range*	— — —	114 (105 -122)^#^/ 41 (33 - 42) ^#^	10 (2 - 19)^#^/2 (1-10)^#^

Area under receiver operating characteristic curve (AUC) and 95% confidence intervals. The second local radiomics model was tested in the leave-one-out cross-validation, thus no results for the training cohort are shown and the validation AUC is the average over the folds. *Most frequently chosen features over different folds. ^#^Median and range of the number of sub-volumes analyzed over different folds.

## Discussion

HNSCCs, exhibiting a high heterogeneity in CT images, were previously shown to respond worse to radiochemotherapy (20–24). Several prognostic radiomic signatures have been proposed recently, but studies on predictive signatures with potential impact on treatment optimization are scarce (31). In this study, we were able to differentiate intratumoral levels of radiosensitivity by means of CT radiomics. Further, we proposed an algorithm for pretreatment detection of radioresistant regions.

Identification of treatment resistant tumor sub-volumes by means of medical imaging has been previously investigated, mainly by detection on hypoxic imaging but also different imaging modalities. Tumor hypoxia is a known adverse prognostic factor for local control after radiotherapy of HNSCC (32). However, only two studies tried to correlate spatial location of tumor recurrence and initial hypoxic region, and only one of them showed overlap of those regions (33, 34). A correlation of high FDG uptake parts of a tumor to regions of more resistant tumor sub-volumes leading to recurrences has been shown (35, 36). Accordingly, FDG-based dose escalation strategies have already been exploited and proven to be feasible (37). However, also other imaging modalities like diffusion-weighted MRI or dynamic contrast-enhanced MRI, were shown to be able to identify risk factors for worse outcomes in head and neck cancer (38). Comparison studies have shown that the volumes defined by FDG-PET versus DWI – MRI do not overlap completely and identify distinct volumes within the primary tumor (39, 40).

Recently, Beaumont et al. (25) correlated location of tumor recurrence to pretreatment local texture features in FDG PET imaging in a small cohort of 15 patients. The performance of their model was comparable to ours (median AUC of 0.71). However, in contrast to our study, their results were only tested in leave-one-out cross-validation, owing to a small number of patients. In addition, the study did not clearly define if CT information was used together with PET information for definition of the primary tumor contour. Delineation based solely on PET images may lead to neglecting tumor regions with low FDG uptake (41). Our local radiomics results indicate that sub-volumes with higher CT heterogeneity are more radioresistant, which is in agreement with previous studies showing that higher tumor-wide CT heterogeneity is linked with reduced local control rate (20–24). Local radiomics in PET/CT has also been investigated in the context of outcome prediction in nasopharyngeal cancer, showing a higher prognostic value than with models based on entire tumor volume analysis (26). However, this study did not use local radiomics for sub-regional detection of treatment resistance sub-volumes.

With the introduction of intensity modulated radiation therapy (IMRT) the concept of delivery higher doses to head and neck tumors has regained interest. IMRT allows not only for better sparing of OARs, but also enables delivery of simultaneously higher doses to selected areas (42), so called dose painting. The identification of radioresistant tumor sub-volumes that require higher radiation doses, is an unmet clinical need. The ESCALOX study currently investigates a dose escalation up to 80.5 Gy using a simultaneous integrated boost (SIB) to the whole primary tumor and large involved lymph nodes (43), whereas another group performed a planning study with dose escalation only to hypoxic areas within the tumor as defined by ^18^F-Fluoroazomycin arabinosid (FAZA) PET/CT and found it to be feasible while respecting the maximum OAR constraints (44). Resistance of head and neck cancer cells to radiotherapy is not conditioned by one single biological feature but rather driven by several different mechanisms (39). Consequently, a method capturing all these distinct features is desirable. The underlying biology for the distinct radiomics signature is not simple nor exactly known for the individual case. But, a correlation of radiomic signature with underlying genomic alterations and biological phenotype has been shown (45). In our study, the selection of the radioresistant sub-volumes is meant to be done by radiomics analysis, which does not account for any particular biological background/histopathological difference. However, a strong relationship between medical images, or more precisely, the extracted, quantitative imaging features, and the underlying tumor phenotype and biology was shown previously (46, 47). Thus, in contrast to the above mentioned study, which selected the radioresistant parts solely based on hypoxic regions, our radiomics approach covers a broader spectrum of underlying biological phenotypes/alterations. Still, the histopathological factors behind the selected sub-volumes in our study are unknown, and thus definition of an adequate dose boost is not straightforward and should be a subject of further research.

This study was performed on relatively small patient cohort. However, for two out of three proposed models (bi-regional and local radiomics with fixed number of sub-volumes), a successful model validation in *a priori* defined cohort was performed. Remarkably, the performance of the models was higher in the validation cohort, in comparison with the training cohort. The validation cohort is a prospective cohort with standardized imaging and treatment protocol – in comparison to the training cohort, which was retrospectively collected. Therefore, higher model performance in the validation might be a result of premature training or better data quality. In the head and neck region, HPV status is known to influence CT values distribution within primary tumor (48). Unfortunately, HPV status was unknown for a large proportion of patients (35%). Consequently, an analysis on HPV status as an effector is not possible due to the high number of missing values in the individual patient cohorts. The analyzed cohort is also heterogeneous in terms of tumor subsites, and theirs impact on the performance of the model should be further investigated. The presence of artifacts did not allow for a full validation of the fixed grid size local model. With the introduction of iterative metal artifact reduction reconstruction algorithms in CT few years ago, this limitation of the model is addressed for future studies (49). Availability of larger data collections may also permit for testing different settings in the local radiomics analysis, such as variable size of the grid or smaller value of the translation vector, allowing for an overlap between sub-volumes. Today, it is not clear which methodology provides optimal results. In our study, we assumed that all of the analyzed tumor sub-volumes are independent and thus, no overlap was allowed. Additionally, classification was performed using logistic regression. In the future, segmentation algorithms may be tested to improve the predictive power of local radiomics.

Additional validation of the proposed approach may be derived from surgical cohorts, where pretreatment local radiomics maps may be correlated with full-mount tumor histopathology. Alternatively, if contrast-enhanced CT is present at the time of recurrence similarity of the radiomics features in the corresponding areas in the two, sequential investigations could be measured to evaluate if the signature remains stable over time.

This study has an inherent selection bias, since only patients with observed recurrences were analyzed. In the real pretreatment classification, a standard radiomics analysis can be used prior to local radiomics to preselect patients with high risk of tumor recurrence, as shown by other studies (20–24).

In conclusion, this is the first study indicating that tumor radioresistance can be localized on pretreatment CT images with validation of the radiomics model in an independent cohort. This is a proof of concept study, indicating that local CT radiomics can be used as predictive biomarker in radiotherapy and potential target for dose intensification.

## Data Availability Statement

The raw data supporting the conclusions of this article will be made available by the authors, without undue reservation.

## Ethics Statement

The studies involving human participants were reviewed and approved by Ethics Committee canton Zurich. The patients/participants provided general consent to participate in this study.

## Author Contributions

MB – performed data analysis, supervised implementation of local radiomics, wrote part of the manuscript and reviewed the manuscript. MP - contributed to study design, performed image registration and contouring, wrote part of the manuscript and reviewed the manuscript. OR - provided expertise, contributed to study design, reviewed contours and registration, and reviewed the manuscript. TF, HS, EH-S, LR and LB - performed image registration and contouring, reviewed the manuscript. MS – performed standard radiomics analysis, reviewed the manuscript, AA – implemented local radiomics, reviewed the manuscript. MH, MG and ST-L - provided expertise, contributed to study design and reviewed the manuscript. All authors contributed to the article and approved the submitted version.

## Funding

This project was supported by the Swiss National Science Foundation Sinergia grant (310030_173303). The clinical study used as one of the cohorts was supported by a research grant from Merck (Schweiz) AG. The funder bodies were not involved in the study design, collection, analysis, interpretation of data, the writing of this article or the decision to submit it for publication.

## Conflict of Interest

ST-L reports grants from Swiss National Science Foundation, during the conduct of the study, and grants from Siemens Healthcare, outside the submitted work. MG reports grants from Swiss National Science Foundation, during the conduct of the study, grants from Siemens Healthcare, and grants from Artificial Intelligence in oncological Imaging Network, University of Zurich, outside the submitted work. MH reports grants and personal fees from GE Healthcare, grants from Alfred and Annemarie von Sick legacy, and grants from Artificial Intelligence in oncological Imaging Network, University of Zurich, outside the submitted work.

The remaining author declares that the research was conducted in the absence of any commercial or financial relationships that could be construed as a potential conflict of interest.

## References

[1] Gatta G Botta L Sánchez MJ Anderson LA Pierannunzio D Licitra L. Prognoses and Improvement for Head and Neck Cancers Diagnosed in Europe in Early 2000s: The EUROCARE-5 Population-Based Study. Eur J Cancer (2015) 51(15):2130–43. 10.1016/j.ejca.2015.07.043 26421817

[2] Siegel RL Miller KD Jemal A. Cancer Statistics, 2019. CA: Cancer J Clin (2019) 69(1):7–34. 10.3322/caac.21551 30620402

[3] Pignon J-P Maître Al Maillard E Bourhis J. Meta-Analysis of Chemotherapy in Head and Neck Cancer (MACH-NC): An Update on 93 Randomised Trials and 17,346 Patients. Radiother Oncol (2009) 92(1):4–14. 10.1016/j.radonc.2009.04.014 19446902

[4] Ho AS Kraus DH Ganly I Lee NY Shah JP Morris LGT. Decision Making in the Management of Recurrent Head and Neck Cancer. Head & Neck (2014) 36: (1):144–51. 10.1002/hed.23227 23471843

[5] Leclerc M Maingon P Hamoir M Dalban C Calais G Nuyts S. A dose Escalation Study With Intensity Modulated Radiation Therapy (IMRT) in T2N0, T2N1, T3N0 Squamous Cell Carcinomas (SCC) of the Oropharynx, Larynx nd Hypopharynx Using a Simultaneous Integrated Boost (SIB) Approach. Radiother Oncol (2013) 106(3):333–40. 10.1016/j.radonc.2013.03.002 23541643

[6] Ferreira BC Sá-Couto P Khouri L Lopes M. Biological Dose-escalated Definitive Radiation Therapy in Head and Neck Cancer. British J Rad (2017) 90(1072):20160477. 10.1259/bjr.20160477 PMC5605059 28186838

[7] Lauve A Morris M Schmidt-Ullrich R Wu Q Mohan R Abayomi O. Simultaneous Integrated Boost Intensity-Modulated Radiotherapy For Locally Advanced Head-and-Neck Squamous Cell Carcinomas: II—Clinical Results. Int J Radiat OncologyBiologyPhysics (2004) 60(2):374–87. 10.1016/j.ijrobp.2004.03.010 15380569

[8] Bourhis J Overgaard J Audry H Ang KK Saunders M Bernier J. Hyperfractionated or Accelerated Radiotherapy in Head And Neck Cancer: A Meta-Analysis. Lancet (London England) (2006) 368(9538):843–54. 10.1016/s0140-6736(06)69121-6 16950362

[9] Sher DJ Adelstein DJ Bajaj GK Brizel DM Cohen EEW Halthore A. Radiation Therapy for Oropharyngeal Squamous Cell Carcinoma: Executive Summary of an ASTRO Evidence-Based Clinical Practice Guideline. Pract Radiat Oncol (2017) 7(4):246–53. 10.1016/j.prro.2017.02.002 28428019

[10] Mroz EA Tward AD Pickering CR Myers JN Ferris RL Rocco JW. High Intratumor Genetic Heterogeneity Is Related to Worse Outcome in Patients With Head and Neck Squamous Cell Carcinoma. Cancer (2013) 119(16):3034–42. 10.1002/cncr.28150 PMC3735618 23696076

[11] Thorwarth D. Radiotherapy Treatment Planning Based on Functional PET/CT Imaging Data. Nuclear Med Rev (2012) 15(C):43–7. 10.5603/NMR.2012.0003

[12] Skjøtskift T Evensen ME Furre T Moan JM Amdal CD Bogsrud TV. Dose Painting For Re-Irradiation of Head And Neck Cancer. Acta Oncologica (2018) 57(12):1693–9. 10.1080/0284186X.2018.1512753 30280623

[13] Welz S Monnich D Pfannenberg C Nikolaou K Reimold M La Fougere C. Prognostic Value Of Dynamic Hypoxia PET in Head and Neck Cancer: Results From a Planned Interim Analysis of a Randomized Phase II Hypoxia-Image Guided Dose Escalation Trial. Radiother Oncol J Eur Soc Ther Radiol Oncol (2017) 124(3):526–32. 10.1016/j.radonc.2017.04.004 28434798

[14] Lambin P Rios-Velazquez E Leijenaar R Carvalho S van Stiphout RG Granton P. Radiomics: Extracting More Information from Medical Images Using Advanced Feature Analysis. Eur J Cancer (Oxford Engl 1990) (2012) 48(4):441–6. 10.1016/j.ejca.2011.11.036 PMC4533986 22257792

[15] Aerts HJ Velazquez ER Leijenaar RT Parmar C Grossmann P Carvalho S. Decoding Tumour Phenotype By Noninvasive Imaging Using A Quantitative Radiomics Approach. Nat Commun (2014) 5:4006. 10.1038/ncomms5006 24892406 PMC4059926

[16] Leijenaar RT Bogowicz M Jochems A Hoebers FJ Wesseling FW Huang SH. Development and Validation of a Radiomic Signature to Predict HPV (P16) Status From Standard CT Imaging: A Multicenter Study. British J Rad (2018) 91: (1086):20170498. 10.1259/bjr.20170498 PMC6223271 29451412

[17] Giraud P Giraud P Gasnier A El Ayachy R Kreps S Foy J-P. Radiomics and Machine Learning for Radiotherapy in Head and Neck Cancers. Front Oncol (2019) 9(174). 10.3389/fonc.2019.00174 PMC6445892 30972291

[18] Bogowicz M Vuong D Huellner MW Pavic M Andratschke N Gabrys HS. CT Radiomics and PET Radiomics: Ready for Clinical Implementation? Q J Nucl Med Mol (2019) 63(4):355–70. 10.23736/s1824-4785.19.03192-3 31527578

[19] Tanadini-Lang S Balermpas P Guckenberger M Pavic M Riesterer O Vuong D. Radiomic Biomarkers For Head And Neck Squamous Cell Carcinoma. Strahlenther Onkol (2020) 196(10):868–78. 10.1007/s00066-020-01638-4 32495038

[20] Bogowicz M Riesterer O Stark LS Studer G Unkelbach J Guckenberger M. Comparison of PET and CT Radiomics For Prediction of Local Tumor Control in Head and Neck Squamous Cell Carcinoma. Acta Oncologica (2017) 56(11):1531–6. 10.1080/0284186X.2017.1346382 28820287

[21] Anderson MD Cancer Center Neck Quantitative Imaging Working Group. Investigation of Radiomic Signatures for Local Recurrence Using Primary Tumor Texture Analysis in Oropharyngeal Head And Neck Cancer Patients. Sci Rep (2018) 8(1):1524. 10.1038/s41598-017-14687-0 29367653 PMC5784146

[22] Vallières M Kay-Rivest E Perrin LJ Liem X Furstoss C Aerts HJ. Radiomics Strategies For Risk Assessment of Tumour Failure in Head-and-Neck Cancer. Sci Rep (2017) 7(1):10117. 10.1038/s41598-017-10371-5 28860628 PMC5579274

[23] Leger S Zwanenburg A Pilz K Zschaeck S Zöphel K Kotzerke J. CT Imaging During Treatment Improves Radiomic Models For Patients With Locally Advanced Head And Neck Cancer. Radiother Onco (2019) 130:10–7. 10.1016/j.radonc.2018.07.020 30087056

[24] Bogowicz M Tanadini-Lang S Guckenberger M Riesterer O. Combined CT Radiomics of Primary Tumor and Metastatic Lymph Nodes Improves Prediction of Loco-Regional Control in Head and Neck Cancer. Sci Rep (2019) 9(1):15198. 10.1038/s41598-019-51599-7 31645603 PMC6811564

[25] Beaumont J Acosta O Devillers A Palard-Novello X Chajon E de Crevoisier R. Voxel-based Identification of Local Recurrence Sub-Regions From Pre-treatment PET/CT For Locally Advanced Head And Neck Cancers. EJNMMI Res (2019) 9(1):90. 10.1186/s13550-019-0556-z 31535233 PMC6751236

[26] Xu H Lv W Feng H Du D Yuan Q Wang Q. Subregional Radiomics Analysis of PET/CT Imaging With Intratumor Partitioning: Application to Prognosis for Nasopharyngeal Carcinoma. Mol Imaging Biol (2019) 22(5):1414–26. 10.1007/s11307-019-01439-x 31659574

[27] McGarry SD Bukowy JD Iczkowski KA Unteriner JG Duvnjak P Lowman AK. Gleason Probability Maps: A Radiomics Tool for Mapping Prostate Cancer Likelihood in MRI Space. Tomography (2019) 5(1):127. 10.18383/j.tom.2018.00033 30854450 PMC6403022

[28] Riesterer O Pruschy M Bender S Sharma A Bogowicz M Tanadini-Lang S. Consolidation Cetuximab After Concurrent Triplet Radiochemotherapy+ Cetuximab in Patients With Advanced Head and Neck Cancer: A Randomized Phase II Study. Radiother Oncol (2020) 150:62–9. 10.1016/j.radonc.2020.06.011 32540337

[29] Zwanenburg A Vallières M Abdalah MA Aerts HJ Andrearczyk V Apte A. The Image Biomarker Standardization Initiative: Standardized Quantitative Radiomics For High-Throughput Image-Based Phenotyping. Radiology (2020) 295:(2):328–38. 10.1148/radiol.2020191145 PMC7193906 32154773

[30] Radiomics team USZ. (2020) Z-Rad Documentation. Available at: https://medical-physics-usz.github.io/ (Accessed March 26, 2020).

[31] Ou D Blanchard P Rosellini S Levy A Nguyen F Leijenaar RT. Predictive and Prognostic Value Of CT Based Radiomics Signature in Locally Advanced Head And Neck Cancers Patients Treated With Concurrent Chemoradiotherapy Or Bioradiotherapy And Its Added Value To Human Papillomavirus Status. Oral Onco (2017) 71:150–5. 10.1016/j.oraloncology.2017.06.015 28688683

[32] Vaupel P. Tumor Microenvironmental Physiology and its Implications For Radiation Oncology. Semin Radiat Oncol (2004) 14(3):198–206. 10.1016/j.semradonc.2004.04.008 15254862

[33] Zschaeck S Haase R Abolmaali N Perrin R Stützer K Appold S. Spatial Distribution Of FMISO In Head And Neck Squamous Cell Carcinomas During Radio-Chemotherapy And Its Correlation To Pattern Of Failure. Acta Oncologica (2015) 54: (9):1355–63. 10.3109/0284186X.2015.1074720 26398663

[34] Boeke S Thorwarth D Mönnich D Pfannenberg C Reischl G La Fougère C. Geometric Analysis Of Loco-Regional Recurrences in Relation to Pre-Treatment Hypoxia in Patients With Head and Neck Cancer. Acta Oncologica (2017) 56: (11):1571–6. 10.1080/0284186X.2017.1372626 28891398

[35] Due AK Vogelius IR Aznar MC Bentzen SM Berthelsen AK Korreman SS. Recurrences After Intensity Modulated Radiotherapy for Head and Neck Squamous Cell Carcinoma More Likely to Originate From Regions With High Baseline [18F]-FDG Uptake. Radiother Oncol J Eur Soc Ther Radiol Oncol (2014) 111(3):360–5. 10.1016/j.radonc.2014.06.001 PMC4469149 24993331

[36] Soto DE Kessler ML Piert M Eisbruch A. Correlation Between Pretreatment FDG-PET Biological Target Volume and Anatomical Location of Failure After Radiation Therapy for Head and Neck Cancers. Radiother Oncol J Eur Soc Ther Radiol Oncol (2008) 89(1):13–8. 10.1016/j.radonc.2008.05.021 PMC2684445 18555547

[37] Madani I Duprez F Boterberg T Van de Wiele C Bonte K Deron P. Maximum Tolerated Dose in a Phase I Trial on Adaptive Dose Painting by Numbers For Head and Neck Cancer. Radiother Oncol J Eur Soc Ther Radiol Oncol (2011) 101(3):351–5. 10.1016/j.radonc.2011.06.020 21742392

[38] Ng SH Liao CT Lin CY Chan SC Lin YC Yen TC. Dynamic Contrast-enhanced MRI, Diffusion-Weighted MRI and (18)F-FDG PET/CT for the Prediction of Survival in Oropharyngeal or Hypopharyngeal Squamous Cell Carcinoma Treated With Chemoradiation. Eur Radiol (2016) 26(11):4162–72. 10.1007/s00330-016-4276-8 26911889

[39] Perri F Pacelli R Della Vittoria Scarpati G Cella L Giuliano M Caponigro F. Radioresistance in Head And Neck Squamous Cell Carcinoma: Biological Bases And Therapeutic Implications. Head Neck (2015) 37(5):763–70. 10.1002/hed.23837 24995469

[40] Houweling AC Wolf AL Vogel WV Hamming-Vrieze O van Vliet-Vroegindeweij C van de Kamer JB. FDG-PET And Diffusion-Weighted MRI In Head-and-Neck Cancer Patients: Implications For Dose Painting. Radiother Oncol J Eur Soc Ther Radiol Oncol (2013) 106(2):250–4. 10.1016/j.radonc.2013.01.003 23395065

[41] Purohit BS Ailianou A Dulguerov N Becker CD Ratib O Becker M. FDG-PET/CT Pitfalls In Oncological Head And Neck Imaging. Insights Into Imaging (2014) 5(5):585–602. 10.1007/s13244-014-0349-x 25154759 PMC4195840

[42] Bhide SA Newbold KL Harrington KJ Nutting CM. Clinical Evaluation of Intensity-Modulated Radiotherapy for Head and Neck Cancers. Br J Radiol (2012) 85(1013):487–94. 10.1259/bjr/85942136 PMC3479880 22556403

[43] Pigorsch SU Wilkens JJ Kampfer S Kehl V Hapfelmeier A Schläger C. Do Selective Radiation Dose Escalation And Tumour Hypoxia Status Impact The Loco-Regional Tumour Control After Radio-Chemotherapy Of Head & Neck Tumours? The ESCALOX Protocol. Radiat Oncol (2017) 12(1):45. 10.1186/s13014-017-0776-1 28249612 PMC5333380

[44] Servagi-Vernat S Differding S Sterpin E Hanin F-X Labar D Bol A. Hypoxia-Guided Adaptive Radiation Dose Escalation In Head And Neck Carcinoma: A Planning Study. Acta Oncol (2015) 54(7):1008–16. 10.3109/0284186X.2014.990109 25562382

[45] Zhu Y Mohamed ASR Lai SY Yang S Kanwar A Wei L. Imaging-Genomic Study of Head and Neck Squamous Cell Carcinoma: Associations Between Radiomic Phenotypes and Genomic Mechanisms Via Integration of The Cancer Genome Atlas and The Cancer Imaging Archive. JCO Clin Cancer Inf (2019) 3:1–9. 10.1200/cci.18.00073 PMC6874020 30730765

[46] Colen R Foster I Gatenby R Giger ME Gillies R Gutman D. NCI Workshop Report: Clinical and Computational Requirements for Correlating Imaging Phenotypes with Genomics Signatures. Trans Oncol (2014) 7(5):556–69. 10.1016/j.tranon.2014.07.007 PMC4225695 25389451

[47] Zwirner K Hilke FJ Demidov G Socarras Fernandez J Ossowski S Gani C. Radiogenomics in Head And Neck Cancer: Correlation Of Radiomic Heterogeneity And Somatic Mutations in TP53, FAT1 and KMT2D. Strahlenther Und Onkol Organ Der Deutschen Rontgengesellschaft [et al] (2019) 195(9):771–9. 10.1007/s00066-019-01478-x 31123786

[48] Bogowicz M Jochems A Deist TM Tanadini-Lang S Huang SH Chan B. Privacy-preserving Distributed Learning Of Radiomics To Predict Overall Survival And HPV Status In Head And Neck Cancer. Sci Rep (2020) 10(1):1–10. 10.1038/s41598-020-61297-4 32161279 PMC7066122

[49] Andersson KM Dahlgren CV Reizenstein J Cao Y Ahnesjö A Thunberg PJMP. Evaluation of Two Commercial CT Metal Artifact Reduction Algorithms For Use In Proton Radiotherapy Treatment Planning In The Head And Neck Area. Medical Physics (2018) 45(10):4329–44. 10.1002/mp.13115 30076784

