# Supplementary material for: Targeting Treatment Resistance in Head and Neck Squamous Cell Carcinoma – Proof of Concept for CT Radiomics-Based Identification of Resistant Sub-Volumes
Source: Front Oncol. 2021 May 27;11:664304. doi: 10.3389/fonc.2021.664304 (PMC8191457; doi:10.3389/fonc.2021.664304)
Supplement: Supplementary file 1 [file DataSheet_1.docx]

**Supplementary Material:**

**Targeting treatment resistance in head and neck squamous cell carcinoma – proof of concept for CT radiomics-based identification of resistant sub-volumes**

Bogowicz Marta^1^*, Pavic Matea^1^*, Riesterer Oliver^1,3^, Finazzi Tobias^1^, Garcia Schüler Helena^1^, Holz-Sapra Edna^1^, Rudofsky Leonie^1^, Basler Lucas^1^, Spaniol Manon^1^, Ambrusch Andreas^1^, Hüllner Martin^2^, Guckenberger Matthias^1^, Tanadini-Lang Stephanie^1^

1. Department of Radiation Oncology, University Hospital Zurich and University of Zurich, Zurich Switzerland
2. Department of Nuclear Medicine, University Hospital Zurich and University of Zurich, Zurich, Switzerland.
3. Centre for Radiation Oncology KSA-KSB, Cantonal Hospital Aarau, Aarau, Switzerland.

*contributed equally

Content

[Volume definition 2](#_Toc63360870)

[Radiomic features 3](#_Toc63360871)

[Local radiomics: Distribution of analyzed subvolumes 7](#_Toc63360872)

[References 8](#_Toc63360873)

# **Volume definition**

1. b)

**
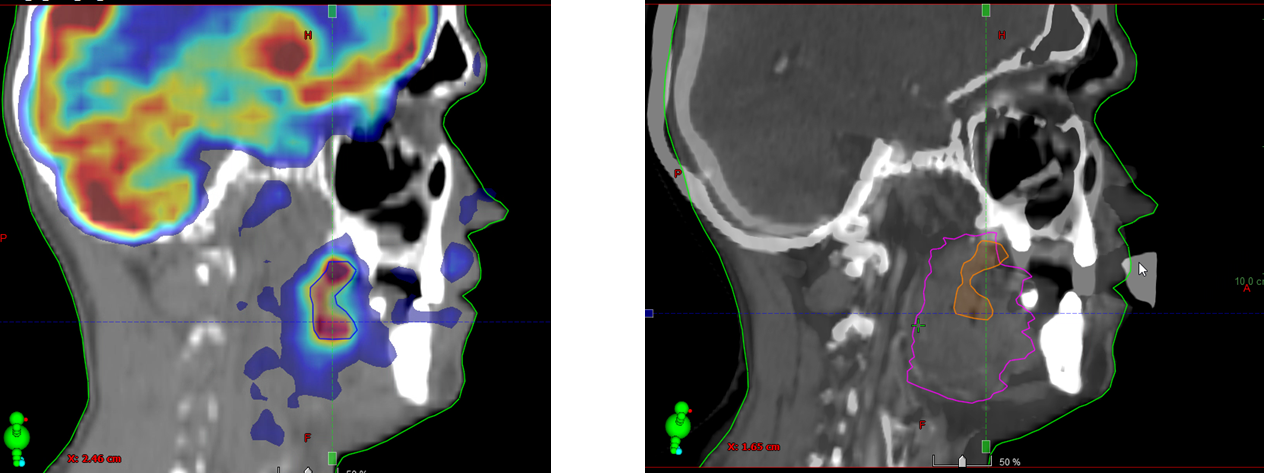
**

Figure S1. Example of recurrence region definition: a) segmentation of recurrence in follow-up FDG-PET/CT, b) registration of pretreatment CT and follow-up FDG-PET/CT, pink – original primary tumor contour, orange – recurrence contour.

| Scanner type  GE MEDICAL SYSTEMS, Discovery LS  GE MEDICAL SYSTEMS, Discovery 690  GE MEDICAL SYSTEMS, Discovery RX  GE MEDICAL SYSTEMS, Discovery STE  GE MEDICAL SYSTEMS, Discovery HR  GE MEDICAL SYSTEMS, SIGNA PET/MR | n = 3  n = 4  n = 7  n = 23  n = 2  n = 1 |
| --- | --- |
| Slice thickness [mm] | 3.27 (2.78 - 4.25) |
| In-plane resolution [mm] | 5.08 (2.34 - 5.47) |
| Administered activity [MBq] | 345.40 (177.50 - 442.19) |
| Delay between administration of FDG and scanning [min] | 58.95 (47.02 - 83.70) |
| Reconstruction algorithm  3D IR  OSEM  VPFXS | n = 30  n = 5  n = 5 |
| Applied corrections  decay corrected  attenuation corrected  scatter corrected  dead time corrected  randoms corrected  sensitivity calibrated using dose calibrator  detector normalization  non-uniform radial sampling corrected | n = 40  n = 39  n = 40  n = 40  n = 35  n = 40  n = 40  n = 7 |

Table S1. PET scanning protocol. For the numerical values median is given together with the range in parenthesis.

# **Radiomic features**

Full list of radiomic features used in this study is presented below. Z-Rad calculates radiomic features (intensity and texture) according to the image biomarker standardization initiative (IBSI, version 9 [1, 2]). Features in the Tab S3 are referenced to appropriate paragraph in IBSI documentation or if feature is not implemented in IBSI details of the calculation are specified.

| radiomics type | matrix | feature name | IBSI reference |
| --- | --- | --- | --- |
| intensity | histogram | minimum |  |
|  |  | maximum |  |
|  |  | mean |  |
|  |  | standard deviation |  |
|  |  | coefficient of variation |  |
|  |  | skewness |  |
|  |  | kurtosis |  |
|  |  | variance |  |
|  |  | median |  |
|  |  | percentile 10th |  |
|  |  | percentile 90th |  |
|  |  | interquartile range |  |
|  |  | range |  |
|  |  | mean absolute deviation |  |
|  |  | robust mean absolute deviation |  |
|  |  | energy |  |
|  |  | entropy |  |
|  |  | root mean square |  |
|  |  | uniformity |  |
|  |  | cumulative volume 20% | Features not specified in IBSI. They refer to a region of interest volume above given threshold T (e.g. 20%) of maximum intensity in this region. Analogous to metabolic tumor volume in PET imaging |
|  |  | cumulative volume 30% |  |
|  |  | cumulative volume 40% |  |
|  |  | cumulative volume 50% |  |
|  |  | cumulative volume 60% |  |
|  |  | cumulative volume 70% |  |
| texture | GLCM | energy |  |
|  |  | entropy |  |
|  |  | contrast |  |
|  |  | correlation |  |
|  |  | homogeneity |  |
|  |  | homogeneity normalized |  |
|  |  | inverse difference |  |
|  |  | inverse difference normalized |  |
|  |  | variance |  |
|  |  | sum of average |  |
|  |  | sum of entropy |  |
|  |  | sum of variance |  |
|  |  | difference entropy |  |
|  |  | difference variance |  |
|  |  | information measures of correlation 1 |  |
|  |  | information measures of correlation 2 |  |
|  |  | maximal correlation coefficient | Feature not defined in IBSI, see [3] |
|  |  | joint maximum |  |
|  |  | joint average |  |
|  |  | difference average |  |
|  |  | dissimilarity |  |
|  |  | inverse variance |  |
|  |  | autocorrelation |  |
|  |  | cluster tendency |  |
|  |  | cluster shade |  |
|  |  | cluster prominence |  |
|  | merged  GLCM | energy |  |
|  |  | entropy |  |
|  |  | contrast |  |
|  |  | correlation |  |
|  |  | homogeneity |  |
|  |  | homogeneity normalized |  |
|  |  | inverse difference |  |
|  |  | inverse difference normalized |  |
|  |  | variance |  |
|  |  | sum of average |  |
|  |  | sum of entropy |  |
|  |  | sum of variance |  |
|  |  | difference entropy |  |
|  |  | difference variance |  |
|  |  | information measures of correlation 1 |  |
|  |  | information measures of correlation 2 |  |
|  |  | maximal correlation coefficient | Feature not defined in IBSI, see [3] |
|  |  | joint maximum |  |
|  |  | joint average |  |
|  |  | difference average |  |
|  |  | dissimilarity |  |
|  |  | inverse variance |  |
|  |  | autocorrelation |  |
|  |  | cluster tendency |  |
|  |  | cluster shade |  |
|  |  | cluster prominence |  |
|  | NGTDM | coarseness |  |
|  |  | contrast |  |
|  |  | busyness |  |
|  |  | complexity |  |
|  |  | strength |  |
|  | GLRLM | gray-level non-uniformity |  |
|  |  | gray-level non-uniformity normalized |  |
|  |  | run length non-uniformity |  |
|  |  | run length non-uniformity normalized |  |
|  |  | short run emphasis |  |
|  |  | long runs emphasis |  |
|  |  | low gray-level run emphasis |  |
|  |  | high gray-level run emphasis |  |
|  |  | short run low gray-level emphasis |  |
|  |  | short run high gray-level emphasis |  |
|  |  | long run low gray-level emphasis |  |
|  |  | long run high gray-level emphasis |  |
|  |  | run percentage |  |
|  |  | gray level variance |  |
|  |  | run length variance |  |
|  |  | run entropy |  |
|  | merged  GLRLM | gray-level non-uniformity |  |
|  |  | gray-level non-uniformity normalized |  |
|  |  | run length non-uniformity |  |
|  |  | run length non-uniformity normalized |  |
|  |  | short run emphasis |  |
|  |  | long runs emphasis |  |
|  |  | low gray-level run emphasis |  |
|  |  | high gray-level run emphasis |  |
|  |  | short run low gray-level emphasis |  |
|  |  | short run high gray-level emphasis |  |
|  |  | long run low gray-level emphasis |  |
|  |  | long run high gray-level emphasis |  |
|  |  | run percentage |  |
|  |  | gray level variance |  |
|  |  | run length variance |  |
|  |  | run entropy |  |
|  | GLSZM | gray-level non-uniformity |  |
|  |  | gray-level non-uniformity normalized |  |
|  |  | size zone non-uniformity |  |
|  |  | size zone non-uniformity normalized |  |
|  |  | small zone emphasis |  |
|  |  | large zone emphasis |  |
|  |  | low gray-level zone emphasis |  |
|  |  | high gray-level zone emphasis |  |
|  |  | small zone low gray-level emphasis |  |
|  |  | small zone high gray-level emphasis |  |
|  |  | large zone low gray-level emphasis |  |
|  |  | large zone high gray-level emphasis |  |
|  |  | zone percentage |  |
|  |  | gray level variance |  |
|  |  | zone size variance |  |
|  |  | zone size entropy |  |
|  | GLDZM | gray-level non-uniformity | Definition of distance differs from IBSI, see [3] |
|  |  | gray-level non-uniformity normalized |  |
|  |  | zone distance non-uniformity |  |
|  |  | zone distance non-uniformity normalized |  |
|  |  | small distance emphasis |  |
|  |  | large distance emphasis |  |
|  |  | low gray-level zone emphasis |  |
|  |  | high gray-level zone emphasis |  |
|  |  | small distance low gray-level emphasis |  |
|  |  | small distance high gray-level emphasis |  |
|  |  | large distance low gray-level emphasis |  |
|  |  | large distance high gray-level emphasis |  |
|  |  | zone percentage |  |
|  |  | gray level variance |  |
|  |  | zone distance variance |  |
|  |  | zone distance entropy |  |
|  | NGLDM | gray-level non-uniformity |  |
|  |  | gray-level non-uniformity normalized |  |
|  |  | dependence count non-uniformity |  |
|  |  | dependence count non-uniformity normalized |  |
|  |  | low dependence emphasis |  |
|  |  | high dependence emphasis |  |
|  |  | low gray-level count emphasis |  |
|  |  | high gray-level count emphasis |  |
|  |  | low dependence low gray-level emphasis |  |
|  |  | low dependence high gray-level emphasis |  |
|  |  | high dependence low gray-level emphasis |  |
|  |  | high dependence high gray-level emphasis |  |
|  |  | gray level variance |  |
|  |  | dependence count variance |  |
|  |  | dependence count entropy |  |

Table S2. Full list of radiomics features used in the study, GLCM – gray level co-occurrence matrix, NGTDM – neighborhood gray tone difference matrix, GLRLM – gray level run length matrix, GLSZM – gray level size zone matrix, GLDZM – gray level distance zone matrix, NGLDM – neighboring gray level dependence matrix

# **Local radiomics: Distribution of analyzed subvolumes**

*
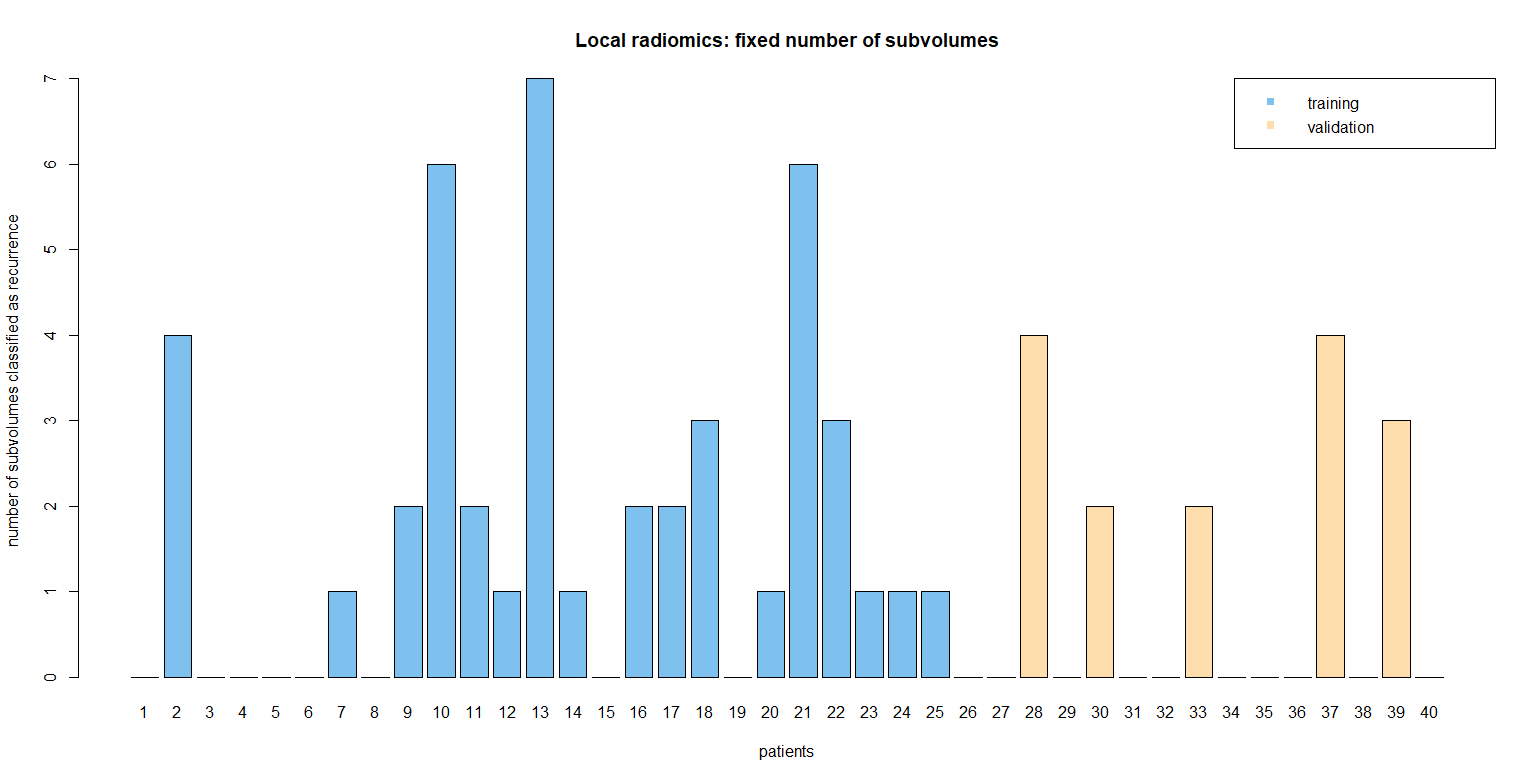
*

Figure S2. Number of subvolumes classified as recurrence in the local radiomics with fixed number of subvolumes. The results are shown on the patient basis. Maximum number of analyzed subvolumes per patient equals 8. Due to placement of the grid or small sizes of recurrences, in some patients no subvolumes were classified as recurrence.


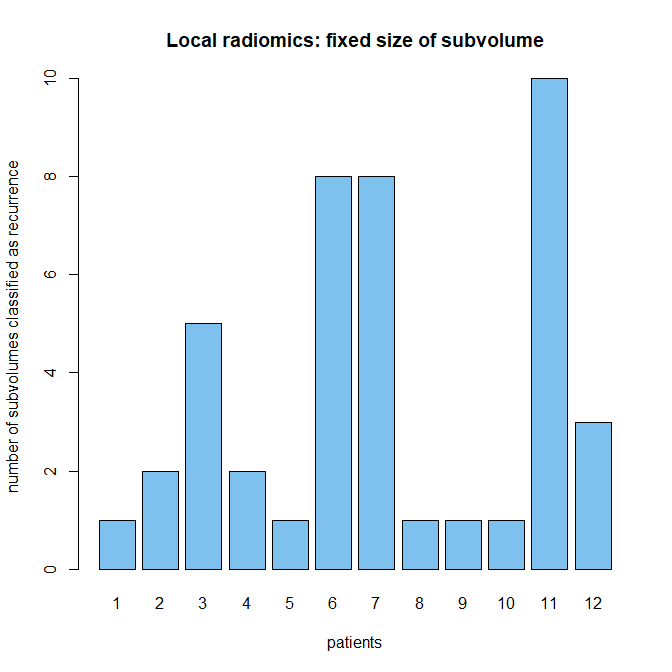


Figure S3. Number of subvolumes classified as recurrence in the local radiomics with fixed size of subvolumes (5x5x5 voxels). The results are shown on the patient basis.

# **References**

1. Zwanenburg A, Vallières M, Abdalah MA, Aerts HJ, Andrearczyk V, Apte A, Ashrafinia S, Bakas S, Beukinga RJ, Boellaard RJR: **The Image Biomarker Standardization Initiative: standardized quantitative radiomics for high-throughput image-based phenotyping**. 2020:191145.

2. **Image biomarker standardisation initiative** [https://arxiv.org/abs/1612.07003v9]

3. **Z-Rad documentation** [https://medical-physics-usz.github.io/]
